# Implementation and use of the Mental Health Gap Action Programme Intervention Guide (mhGAP-IG): A review of the grey literature

**DOI:** 10.7189/jogh.11.04022

**Published:** 2021-04-17

**Authors:** Jessica Spagnolo, Shalini Lal

**Affiliations:** 1 Département des sciences de la santé communautaire, Université de Sherbrooke, Québec, Canada; 2 Centre de recherche Charles-Le Moyne-Saguenay-Lac-St-Jean sur les innovations en santé, Campus Longueuil – Université de Sherbrooke, Longueuil, Québec, Canada; 3 École de réadaptation, Faculté de médecine, Université de Montréal, Québec, Canada; 4 Centre de recherche du Centre hospitalier de l’Université de Montréal, Québec, Canada; 5 Douglas Mental Health University Institute, Montreal, Québec, Canada

## Abstract

**Background:**

The World Health Organization (WHO)’s Mental Health Gap Action Programme (mhGAP)-Intervention Guide (IG) aims to integrate mental health into primary care/community-based settings by equipping non-specialists with tools, training, and support to deliver evidence-based interventions. With the growing popularity of the mhGAP-IG, a systematic review was conducted by Keynejad and colleagues (2018) to identify articles reporting on evidence generated from the implementation and evaluation of the mhGAP-IG in low- and middle-income countries (LMICs). Their review identified peer-reviewed articles and one thesis. In this current review, we report on the implementation and use of mhGAP-IG documented in the grey literature, an important and accessible channel to share information for LMICs.

**Methods:**

We searched grey literature databases for documents that reported on the implementation and/or use of the mhGAP-IG or its training modules: ProQuest Dissertations & Theses Global, the Mental Health Innovation Network (MHIN) database, the WHO website, the mhGAP Newsletter, and the first 10 pages of Google search results. Authors developed and adapted search strategies according to database characteristics. Database searches were completed by November 12, 2019.

**Results:**

One hundred and fifty-one (n = 151) documents were included in our review. We report on where the mhGAP-IG has been implemented and/or used worldwide. Many types of personnel were trained in the mhGAP-IG and/or used it in clinical practice. Contextual barriers and facilitators may influence the implementation and/or use of the mhGAP-IG, and we organized these according to structural, organizational, provider, patient, and innovation characteristics. Some information on evaluating the mhGAP-IG was documented in the grey literature. Outcomes included: feasibility of implementing and/or using the mhGAP-IG, its coverage, its impact on the capacities of personnel, patient outcomes, and policies, as well as program costs.

**Conclusions:**

This review of the grey literature provides rich experiential knowledge that can complement information documented in the peer-reviewed literature. It is important for researchers conducting reviews on global health/global mental health topics to consider incorporating grey literature search strategies in their reviews. This may not only help to acknowledge the research/dissemination realities of many LMICs, but also to generate findings that reinforce and/or expand those documented in peer-reviewed articles.

The World Health Organization (WHO) launched the Mental Health Gap Action Programme (mhGAP) in 2008, which aims to further build and scale-up mental health services in LMICs [1]. Specifically, this programme represents the WHO’s continued commitment to delivering mental health services through integrated intervention packages and addressing pre-existing barriers to mental health care to facilitate package scale-up [1]. For example, the mhGAP provides a framework for scaling-up mental health interventions by providing guidance on political commitment to mental health care, the assessment of needs and resources, the development of a policy and legislative infrastructure, the “how to” of delivering the intervention package, the strengthening of human resources, the mobilization of financial resources, and monitoring and evaluation [1]. This programme is accompanied by guidelines like the mhGAP-Intervention Guide (IG), which aims to further integrate mental health into primary care and community-based settings by equipping non-specialists with tools, training, and support to deliver evidence-based interventions for what the WHO deems priority mental, neurological, and substance use disorders in low- and middle-income countries (LMICs). These include depression, psychoses, epilepsy, child and adolescent mental and behavioural disorders, dementia, problems related to substance use, and self-harm/suicide [2,3]. The mhGAP-IG was first released in 2010 [2] and is currently in its second version [3].

With the growing popularity of the mhGAP-IG, Keynejad and colleagues (2018) [4] conducted a systematic review to identify articles reporting on evidence generated from its implementation and evaluation in LMICs. Their search yielded 32 peer-reviewed articles and one thesis implemented in 20 countries and/or territories [4]. However, we wondered about the available information on the implementation and use of the mhGAP-IG beyond the peer-reviewed literature (ie, the grey literature). Grey literature is defined as “a range of published and unpublished material which is not normally identifiable through conventional methods of bibliographic control” [5]. Such a realm may be an important and accessible channel for LMICs to share information. For example, studies show inequities in research funding allocation. Limited research funding is usually available for countries with most of the global health problems [6]. Studies also show challenges related to the inclusion of authors affiliated with or from LMICs in peer-reviewed publications [7-10]. Hence, publishing in peer-reviewed journals may be influenced by Global North funding and collaborations. As such, it may be more feasible to disseminate evidence on implementing and using the mhGAP-IG through grey literature sources, such as via blog posts, newsletters, and/or internal reports. Moreover, given the novelty of the mhGAP-IG [2] and its updates [3], as well as the 2017 transfer of its manualized content to an electronic version [11], information on its implementation and use may not yet be published in academic or traditional bibliographic realms.

To our knowledge, the grey literature is a realm previously unexplored for information on the mhGAP-IG. With this paper, our goal was therefore descriptive: to search beyond the peer-reviewed literature to explore and report information about the implementation and use of the mhGAP-IG.

## METHODS

We conducted a review of the grey literature. Aligned with previous studies and methodologies used for conducting a systematic review of the grey literature [12], we first developed a detailed plan for conducting the review, documented in the form of a review protocol. This protocol included the rationale for conducting the review, the search terms, the websites, the limits to the search, the inclusion and exclusion criteria, and a plan for data extraction.

### Information sources and search strategies

We searched the following grey literature databases: ProQuest Dissertations & Theses Global, the Mental Health Innovation Network (MHIN) database, the WHO website, the mhGAP Newsletter, and the first 10 pages of Google search results. The search strategies were developed by Author 1 and Author 2 and they were adapted according to the characteristics of the databases. Databases were searched starting on 17 September 2019 and they were completed on 12 November 2019.

#### Proquest Dissertations & Theses Global

ProQuest Dissertations & Theses Global was included as part of our information sources because of recent increases in the support allocated to global mental health training [13]. This database regroups students’ final work, which may include any research done on the mhGAP-IG’s implementation and use in LMICs. ProQuest Dissertations & Theses Global was searched using the keywords “Mental Health Gap Action Program*” and its abbreviation “mhGAP,” which also serves to target documentation on the IG. The keywords were entered within the Search Fields section of the interface. Both keywords were combined with OR to generate the maximum results. We decided to include only these two keywords to keep the search focused on content directly related to the mhGAP.

#### Mental Health Innovation Network

The MHIN [14] regroups activities, programs, and/or initiatives in global mental health that aim to improve the lives of those living with mental, neurological, and substance use disorders [14]. The MHIN’s team is based at the Centre for Global Mental Health at the London School of Hygiene and Tropical Medicine (UK), and WHO’s Department for Mental Health and Substance Abuse (Geneva, Switzerland) [14]. In 2017, the MHIN had more than 3800 members, 232 organizations, and a plethora of resources and contributions (ie, blog posts, manuals, webinars, research, and podcasts) [15]. These resources and documentation may therefore not be reflected in peer-reviewed literature. The MHIN was searched using three keywords, entered one at a time in the Search section of the interface. Keywords include: “Mental Health Gap Action Programme,” “mhGAP,” and “Mental Health Gap Action Program.”

#### WHO website

The WHO website [16] was searched because it groups information specifically on the mhGAP and its accompanying intervention tools, such as the mhGAP-IG. A component of the WHO website is the mhGAP Newsletter [17], a newsletter produced to describe ongoing mhGAP activities. When mhGAP activities are being implemented in LMICs, the Department of Mental Health and Substance Abuse at the WHO Headquarters in Geneva aims to cover experiences with such interventions, and the impact they may have on target beneficiaries. The newsletter stories are disseminated globally by email to subscribers, as well as on the WHO website [17]. The WHO website was searched in two ways. First, the mhGAP newsletters section was retrieved by typing “mhGAP Newsletters” in the search section of the WHO website. At the time of the search (between 17 September 2019 and 12 November 2019), 19 editions of the mhGAP newsletter were listed on the WHO website, ranging from January 2011 to April 2019. Each of these newsletters were searched manually. The WHO website was also searched using the keyword “Mental Health Gap Action Program” in the search section of the website.

#### Google

We searched the first 10 pages of Google search results for relevant documents and/or information on the implementation and use of the mhGAP-IG. Google is a common search engine for grey literature reviews, and we searched the database using three keywords, as follows: “Mental Health Gap Action Programme” OR “mhGAP” OR “Mental Health Gap Action Program.” Keywords were combined with OR to generate the maximum results.

### Document selection, data extraction, and analysis

Data selection, data extraction, and analysis were primarily conducted by Author 1, and both process and outcomes were reviewed and discussed at several stages with Author 2. First, all documentation generated by the search strategy was included in a data extraction file using Excel software, each tab regrouping the results for each of the databases included. The first author and a research assistant conducted the search strategy. They then removed the duplicates in each of the tabs. Second, the first author applied the inclusion criteria to each of the items generated by the search strategy. Inclusion criteria were documentation that described the implementation and/or use of the mhGAP-IG. We also included: documentation that mentioned the mhGAP with for example the words training, guidelines, or that related to capacity-building and documentation that referred to modules of the mhGAP-IG; as well as documentation that highlighted prospective use. We excluded peer-reviewed literature that we found through our grey literature search. If results met these eligibility criteria, they were included in the paper. There were six documents that the first author was unsure about (ie, labelled as ‘maybe’; these were then reviewed in detail by the second author, as part of the decision-making process). This resulted in 151 documents included in the review. Third, for these included documents, the first author extracted and aggregated information related to the following themes: country of implementation; type of personnel who used the mhGAP-IG and in what setting; mhGAP-IG modules used; information on mhGAP-IG adaptation; contextual factors that may influence the implementation and/or use of the mhGAP-IG; and information on type of outcomes used to assess mhGAP-IG’s implementation and impact. These categories were used to organize the results section of the paper.

## RESULTS

**Figure 1:**
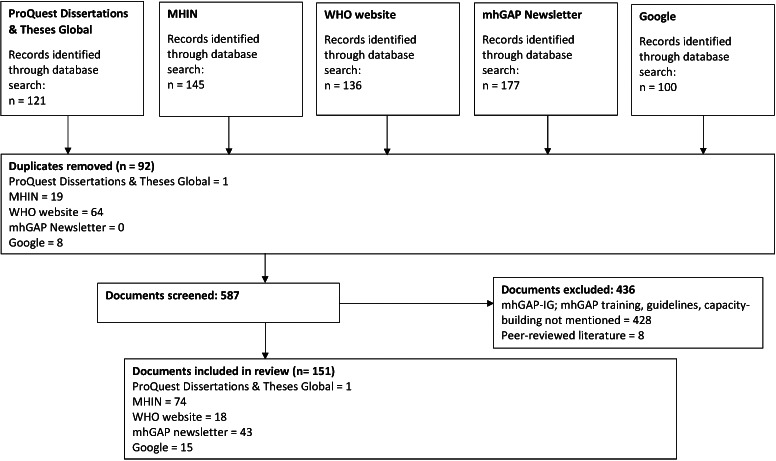
Flowchart identifying documents to be included in the review (Adapted PRISMA Flow Diagram). MHIN – Mental Health Innovation Network, WHO – World Health Organization, mhGAP – Mental Health Gap Action Programme, mhGAP-IG – Mental Health Gap Action Programme Intervention Guide.

The search and selection process are described in **Figure 1**. One hundred and fifty-one (n = 151) documents were retained. A full list of included documents may be found in the Table S1 in the **Online Supplementary Document****.**

### Country of implementation

The documentation we reviewed pertained to the implementation and/or use (including prospective use) of the mhGAP-IG in 92 countries/territories, primarily countries classified as LMICs according to the World Bank’s Income Categories [18]. Specifically, our findings show that 18.68% of countries implementing and/or using the mhGAP-IG are classified as low-income, 31.87% as lower-middle-income, 31.87% as upper-middle-income, and 17.58% as high-income. To generate these percentages, we relied on a denominator of 91 countries/territories, as one country/territory included in our review was not listed in the World Bank Income Categories [18]. In addition, our findings show that most countries implementing and/or using the mhGAP-IG according to the grey literature were those of the WHO Region of the Americas (33.33%). Other countries were represented as follows: 28.74% from the WHO African Region; 11.49% from the WHO Eastern Mediterranean Region; 11.49% from the WHO European Region; 8.05% from the WHO South-East Asia Region; and 6.90% from the WHO Western Pacific Region. To generate these percentages, we relied on a denominator of 87 countries/territories, as five countries/territories included in our review were not identified on the website for their respective WHO Region [19]. A full list of countries identified by our review is included in the Table S2 in the **Online Supplementary Document**.

Of note, in one mhGAP Newsletter (June 2014), there was mention of six Caribbean countries and 14 countries of the WHO Region of the Americas, yet the specific countries were not explicitly listed. Similarly, one retrieved document from the WHO website and one from Google mentioned 14 Caribbean and 13 Latin American countries [20], yet these were not explicitly listed. These countries may have been omitted from the country list (Table S2 in the **Online Supplementary Document**). The same applies to the five Francophone and Anglophone countries listed but not explicitly mentioned in one Google document [20].

### Types of users and settings

The mhGAP-IG was used as a training tool and/or in clinical practice to help personnel detect, treat, and manage mental, neurological, and substance use disorders, as well as refer people presenting with these conditions to specialists. The following types of personnel were engaged with the mhGAP-IG, either in training and/or by using it in clinical practice: 1) national entities like officials from the Ministry of Health, national network managers, and staff from the District of Health; 2) non-specialists like nurses, primary care physicians, physician assistants, resident physicians (for example, emergency medicine trainees), social workers, paramedics, public safety officers, community workers, auxiliary staff, community health extension workers, midwives, aid workers, and human immunodeficiency virus (HIV) lay counselors; 3) specialists like psychologists, psychiatrists (some of which then became trainers), mental health nurses, mobile mental health teams, community mental health workers, psychosocial workers, and pediatricians; 4) health planners and administrators including directors of nursing, community welfare officers, and chief welfare officers; and 5) community champions/leaders, like health and nutrition committee members, traditional birth attendants, community resource persons, volunteers, mothers, family members of children affected by mental health problems, teachers, religious leaders, and traditional healers. There was also mention of the mhGAP-IG training of master trainers to further the implementation process.

In terms of settings, the mhGAP-IG was delivered in primary care and community-based settings like primary care clinics, family medicine centers, as well as in the prison system, hospitals (district, provincial, and teaching, as well as outpatient facilities), and emergency/conflict settings like refugee camp health facilities. The mhGAP-IG was also offered in formal education settings: for example, by integrating its modules into medical undergraduate curricula at universities (Ethiopia, Somalia). In another example, medical students from the United Kingdom were paired with medical students from India and Somaliland to e-learn the mhGAP-IG modules, promoting peer-to-peer learning across cultures [21,22]. In addition, the mhGAP-IG was incorporated into the teaching curricula to train students in Nepal by didactic video lecture [23] and to train community mental health workers in Haiti through a program called Psy-pour-Haïti [24]. The mhGAP-IG was also included in leadership programs as part of continuing education. For example, in Libya, primary mental health care officers were offered a newly minted six-month diploma programme that included modules of the mhGAP-IG. The University of Ibadan in Nigeria coordinates the Mental Health Leadership and Advocacy Programme (mhLAP) where mental health leaders, officials, and advocates from The Gambia, Ghana, Liberia, Nigeria, and Sierra Leone can further their mental health learning, including of the mhGAP-IG. Through this programme, for example, national master trainers could be trained to use the mhGAP-IG, after which they would train non-specialists in their respective countries.

### mhGAP-IG modules

mhGAP-IG modules that were used in the documentation that we reviewed include: the general introduction to the mhGAP-IG; general principles of care; epilepsy; developmental and behavioural disorders in childhood and adolescence; conditions specifically related to stress (acute stress, posttraumatic stress); dementia; depression; child mental health; psychosis; schizophrenia; problems related to alcohol and drug use; self-harm/suicide; and other significant, unexplained emotional or medical complaints. Use of the mhGAP implementation tools and the operations manual was mentioned. Beyond the mhGAP-IG (but included in complementary training), some trainees received courses on psychotherapeutic interventions on cognitive behavioural therapy, family therapy, and counseling skills.

### mhGAP-IG adaptations

The mhGAP-IG along with its accompanying training material (ie, PowerPoints, facilitator and participant guides) are standardized documents that the WHO suggests adapting prior to use [2,3]. In the documentation that we reviewed, there was mention of mhGAP-IG contextualization to the local context (ie, translation to local languages and/or adaptation to meet realities of primary and community-based health care settings, affected displaced populations and host communities, or humanitarian settings) in 31 countries (Table S2 in the **Online Supplementary Document**). Some initiatives mentioned that adaptations were made to specific mhGAP-IG training modules and their accompanying training materials so that they fit into specific time frames and/or so that the mhGAP-IG could be used via online platforms.

### Contextual factors influencing implementation and use

The mhGAP-IG was implemented and used within specific contexts. Contextual factors that may have influenced the implementation and use of the mhGAP-IG were highlighted in some of the documentation that we reviewed. We organized examples of these factors according to the following categories: structural (ie, the sociocultural context in which an organization is nested), organizational (ie, aspects related to the organization in which an innovation like the mhGAP-IG is implemented), provider (ie, aspects related to the provider that implements the innovation within the organization), patient (ie, aspects related to the people exposed to the innovation, such as health beliefs, personality traits, etc.), and innovation (ie, aspects related to the innovation to be implemented, such as quality, usefulness, etc.). These categories were inspired by Chaudoir and colleagues (2013)’s framework [25]. **Table 1** illustrates examples of the barriers and facilitators to mhGAP-IG implementation and/or use.

**Table 1:** Contextual factors (barriers and facilitators) that may influence mhGAP-IG implementation and use

Factors	Barriers	Facilitators
**Structural**	***Political***	***Political***
	- political turnover (ties with specific political leaders may have encouraged mhGAP-IG implementation and use)	- support from and collaborations with the Ministry of Health and/or other national entities
	- challenging working relationships with certain policymakers and government staff	- new mental health legislation (or strategies) to further encourage funding for mental, neurological, and substance use disorders and health system strengthening in primary care and/or community-based settings
	- high conflict, political tension, humanitarian crisis (ie, displacement)	- the revision of national mental health treatment protocols
	- financial crisis	- the creation of a national mental health committee at the level of the Ministry of Health
	- political tension	- the inclusion of mhGAP-IG training modules as part of the national medical training curricula
	- limited funds allocated to mental health	- national workshops to disseminate pilot findings of mhGAP training, which can encourage scale-up
	- no (or outdated) mental health plans to address mental, neurological, and substance use disorders (if a strategy is available, it may not be adopted and/or operationalized)	
	- centralized systems of care (challenging reach of mental health services)	
	***Infrastructure***	***Infrastructure***
	- under-funded and limited facilities (including community organizations)	- leveraging available human resources and care settings, from primary to specialized care
	- fragmented services	
	- poorer quality of health care services	
	- difficulty accessing health care facilities (eg, persistent conflict)	
	- limited human resources (including mental health specialists to provide non-specialists with support)	
	- lack of a national committee for implementing the mhGAP training	
	- limited and/or high cost of medications	
	- inconsistent and/or unavailable Internet coverage (for mhGAP-IG implementation and use that rely on virtual tools)	
	***Socio-cultural***	***Socio-cultural***
	- stigma against mental illness	- community mental health awareness
	***Geographical***	
	- difficult terrain to access clinics	
	- natural disasters (ie, earthquakes, typhoons) making physical access to care more difficult	
**Organizational**	***Infrastructure***	***Infrastructure***
	- understaffing at health facilities where training was implemented	- planning for sustainable support and supervision mechanisms
	- frequently out-of-stock medication within health care clinics	- developing an appropriate referral system to coordinate care
	- lack of appropriate space and privacy for mental health care delivery	- defining roles and responsibilities for all stakeholders involved in the mhGAP-IG implementation
	- questionable fidelity of data collected due to poorer documentation, limited electronic data collection systems, and time-consuming nature of data reporting and collection	- training many types of non-specialists within the health care centers
	- lack of supervision or support to implement the mhGAP training in health care centers	- providing mhGAP-based training in health care centres with available medication
		- leveraging already used technology to participate in training and to treat patients
		- employing a cascade model of training
	***Collaborations***	***Collaborations***
	- insufficient intersectoral collaboration	- receiving support from key organizations (WHO, PAHO), universities, non-governmental organizations, psychiatrists – to facilitate regular supervision and ongoing feedback to trainees)
	- difficulties in the referral processes between organizations, including challenges with continuing of care – from specialized settings to community follow-up	
	***Vision***	***Vision***
	- low mental health priority in health care centres	- fostering a vision that adopts evidence-based services
	- limited mental health leadership within health care centres, affecting providers’ engagement in mental health care delivery and their use of the mhGAP-IG	
**Provider**	***Non-specialists***	***Non-specialists***
	- heavy workload, with competing priorities in daily practice beyond mental health care	- non-specialists’ willingness to include mental health as part of routine clinical tasks
	- professional attrition (retirement, promotion, transfers)	- a reliance on and continuous use of feedback
	- negative bias against some treatment interventions listed in the mhGAP-IG	- intervention ‘buy-in’
	- staff rotation	- an emphasis on training newly graduated professionals to further foster acceptance of mental health care delivery in primary care and/or community-based settings
	***Mental health specialists***	
	- reluctance to recognize non-specialists’ role in mental health care	
**Patient**	- experiences of mental health stigma	- satisfaction with services received by trained staff
**Innovation**	- unavailability of accompanying training materials in local languages	- adaptability of the mhGAP-IG and accompanying training tools to local contexts and needs
	- omitting to implement the supervision component post-mhGAP training	- the training’s clinical utility (including interactive components like group discussions and role plays) and user-friendliness
	- not enough mental health training to meet all mental health learning gaps	- relying on technology to provide training

mhGAP-IG – Mental Health Gap Action Programme Intervention Guide, mhGAP – Mental Health Gap Action Programme, WHO – World Health Organization, PAHO – Pan-American Health Organization

Of note, the Pan-American Health Organization (PAHO)’s Virtual Campus for Public Health (VCPH) was described as facilitating training on using the mhGAP-IG. This platform provides opportunities for synchronous and asynchronous learning on many diverse topics related to public health, including the mhGAP-IG [26]. This innovative way of teaching the mhGAP-IG was conducted in Caribbean countries, as well as in Latin American countries like Guyana. With this platform, trainees could learn at their own pace, as well as connect with other participants and instructors. In Mexico, combined periods of distance learning and in-person seminars were also employed. In addition, Colombia used the VCPH to group the expertise of four universities to teach the mhGAP-IG. The WHO Collaborating Centre for Mental Health Services Research and Training from Spain’s *Universidad Autónoma de Madrid* (UAM) Department of Psychiatry also used this online platform to provide mhGAP-based training in Mexico. The VCPH content is available in English, French, Spanish, and Portuguese [26].

Beyond the VCPH, training and/or clinical support to non-specialized mental health care staff was provided using other technological means. Some examples include the use of didactic videos to provide lecture-based training of mhGAP-IG content in Nepal [23]; a mobile technology to train, supervise, support, and monitor application of the mhGAP-IG’s depression module in Kenya [27]; an interactive voice response and an avatar-assisted training and supervision system related to developmental delay for families and volunteers in Pakistan (The FaNs for Kids Project, a pilot project for sustainable scale-up [28]); a mobile-based clinical decision support tool based on the mhGAP-IG’s algorithm to help staff identity and manage mental disorders, and a mobile follow-up for trainees in India (SMART Mental Health) [29]; and Skype for mhGAP-IG trainee supervision in Nigeria and offered by Global North partners. Other examples included cell phone call reminders for community health workers involved in learning the mhGAP-IG and the provision of video-conferencing and educational CDs to support self-learning training in Afghanistan; tablets provided during the mhGAP-IG training to further interactive learning and their use in clinical practice in the Union of Comoros; the mobile version of the mhGAP-IG child and adolescent depression module to support staff providing care in South Africa and Zambia [30]; and the electronic version of the mhGAP-IG on a mobile device for routine use in community-based settings in Nepal and Nigeria (Emilia project) [31,32].

### mhGAP-IG evaluation

Some documents we reviewed mentioned outcome data. We opted to qualitatively report on examples of the types of outcomes mentioned in the documents that we reviewed, given that outcome data was reported heterogeneously across initiatives. These outcomes are summarized in **Table 2**.

**Table 2:** Outcome measures for the mhGAP-IG implementation and use, with associated examples and tools, if mentioned

Evaluation outcomes	Examples	Tool (if mentioned)
**Feasibility**	Developing and implementing an mhGAP-based training and/or mental health care plans including the mhGAP-IG	
	Implementing and using the mhGAP-IG (electronic vs paper versions)	
	- staff acceptability	
	- non-specialists’ ability to provide evidence-based treatment according to patient symptoms	
	- people with depression being identified by non-specialists	
	- affordability and cost-effectiveness	
	- level of clinical support and supervision to trainees	
	- level of mental health stigma among non-specialists	
**Coverage**	Number of personnel trained to use the mhGAP-IG	
	Number of people who sought care by trainees or personnel using the mhGAP-IG in clinical practice	
	Availability of evidence-based services at facilities, including those based on the mhGAP-IG	
	Treatment gap, measured pre- and post-mhGAP-IG implementation	
	Treatment coverage using the e-mhGAP-IG and the paper version	
	Number of personnel per health care facility who learned to use the mhGAP-IG	
	Number of personnel who received support and supervision sessions by specialists	
	Availability of psychotropic medication supply	
	Number of prescriptions including by mhGAP-IG trainees	
	Healthcare utilization by families	
	Level of mental health integration in primary care sites including organizational-level integration of mental health, to make care more accessible	International Medical Corps Primary Health Care Integration Checklist
**Impact on personnel**	Mental health knowledge	mhGAP knowledge questionnaire
	Mental health attitudes	MICA; SDS; IAT
	Mental health confidence	
	Mental health self-efficacy	
	Self-reported mental health practice, changes in practice, enhancement of skills	
	- screening, accuracy of detection	
	- diagnosis	
	- patient treatment and management, including prescribing and offering psychosocial care	
	- referrals	
	Therapist and non-specialist competence	ENACT scale; TASC-R
	Satisfaction with training (feedback about the program)	
	Quality checks	On-the-job supervision checklists; Institutional quality checklists, including verification that appropriate records are being kept for each patient, and a review of client diagnosis and care plans; Quality Improvement measures
**Impact on patient outcomes**	Mental health conditions treated by personnel	
	Costs (travel costs for people accessing care and treatment costs for families)	
	Quality of life	WHO Quality of Life-BREF; European Quality of Life Scale
	Disability	WHO-DAS; WHO-DAS-CHILD
	Patients’ integration back into the community	
	Experience of stigma	DISC-12
	Patient follow-up (returning to appointments)	
	Functioning, overall symptoms, well-being	HSCL; HTQ; GHQ-12
	Depressive symptoms	BDI; ZLDSI; PHQ-9
	Number of seizures and their level of severity	
	Suicidal thoughts	
	Symptom remission and recovery	Health of Nations Outcomes Scale; Qualitative interviews: people with lived experiences, family caregivers, health care providers, community leaders
	Socio-emotional well-being of children	
	Impact on children’s families (stigma and parental distress, utilization of health care services)	
	Patient satisfaction	Verona Service Satisfaction Scale; mhGAP training reports
	Decrease in mortality (eg, by suicide)	
**Impact on policies**	Psychotropic medication supply	
	Annual mental health budget allocation (eg, transition from a previously WHO-supported drug treatment and care system to one that is locally funded)	
	Improvements in human resources	
	Creation of a health information system for depression and other medical conditions	
	Political interest in mental health (eg, the establishment of a mental health unit at the Ministry of Health and the launching of national mental health policies)	
**Program cost**	Cost of implementing the mhGAP-IG (including offering the training)	
	Economic analysis (eg, costs of mental health treatment from specialists vs non-specialists)	
**Community impact**	Community mental health literacy including being able to recognize mental, neurological, and substance use disorders, as well as mental health awareness)	
	Proactive case finding	Community Informant Detection Tool
**Process of implementing**	Barriers and enablers to implementation	Focus groups and in-depth interviews

mhGAP-IG – Mental Health Gap Action Programme Intervention Guide. MICA – Mental Illness: Clinicians’ Attitudes, SDS – Social Distance Scale. IAT – Implicit Association Tests, ENACT – Enhancing Assessment of Common Therapeutic, TASC-R – Task-Sharing Adherence and Specific Competency Rating Scale, WHO – World Health Organization, DAS – Disability Assessment Schedule, DISC-12 – Discrimination and Stigma Scale, HSCL – Hopkins Symptom Checklist, HTQ – Harvard Trauma Questionnaire, GHQ-12 – General Health Questionnaire, BDI – Beck Depression Inventory, ZLDSI – Zanmi Lasante Depression Symptom Inventory, PHQ-9 – Patient Health Questionnaire

## DISCUSSION

As shown by our review of the grey literature, we identified over 90 countries in which the mhGAP-IG was implemented and/or used, including for its prospective use. Our review highlights the wide span of the mhGAP-IG’s implementation and use and shows a plethora of experiential knowledge available from the grey literature, including news articles about peer-reviewed papers [33]. While the details provided in the documentation found were variable and may be limited in some cases, many of the documents we reviewed provided valuable information. Our review highlights the types of mhGAP-IG modules and personnel who use the mhGAP-IG. For example, we identified a wide range of modules being used, from modules on common mental disorders (depression, anxiety) to conditions considered more severe and complex (dementia, psychosis, alcohol and substance use disorders, self-harm/suicide). Training in such mental, neurological, and substance use disorder modules considered more severe and complex may be a way to help address mental health stigma [4,34-36], which was identified in the documents and can be a contextual factor that challenges the implementation and use of the mhGAP-IG. We also found that several types of personnel participated in mhGAP-IG training and/or used it in clinical practice, most being non-specialists in mental health care. This range of personnel who may be working in different settings highlights the collaborative approach that may be favoured in the mhGAP-IG to offer care [2,3]. It also emphasizes the importance of task-sharing, the increased involvement of non-specialists in mental health care and with the support and supervision of specialists [37,38]. One stakeholder group identified by our search was traditional healers. Traditional healers may be consulted for mental health care and might therefore be valuable resources for the detection of mental, neurological, and substance use disorders [39,40]. Our review found that many countries have engaged in contextual adaptations of the mhGAP-IG or accompanying training material. These adaptations are encouraged by the WHO and may be a way to foster local ownership of the mhGAP-IG implementation and use to encourage its scalability [4,41-43].

Our review identified contextual factors that may influence the implementation and use of the mhGAP-IG and examples of the types of outcomes that were considered and/or used to assess its impact. This experiential knowledge may be beneficial to countries interested in mhGAP-IG implementation, especially since this documentation in the grey literature may be more accessible in LMICs. For example, it can highlight practical and policy changes and/or encourage the reinforcement of facilitators that may further support implementation and use of mhGAP-IG guidelines, as well as task-sharing in respective countries. In addition, program implementors and/or evaluators can mobilize our findings from the grey literature to help identify types of outcomes which might not necessarily be included in the mhGAP training packages, in order to assess impact [44]. Our findings also reinforce and expand on evidence related to contextual barriers and facilitators from the peer-reviewed literature, some of which are more largely discussed as factors influencing the integration of mental health and/or mental health programmes into primary care and/or community-based settings [45], one of the mhGAP objectives.

Our findings highlight that some mhGAP-IG initiatives relied on technological tools to support training and/or guideline implementation into clinical practice. In the context of COVID-19, virtual means to provide mental health training and/or deliver mental health services are being encouraged [46]. We can learn to offer virtual mental health training and support to non-specialists on their mental health care delivery from the experiential knowledge gained by mobilizing existing platforms and infrastructure, such as the VCPH and mobile applications like the mhGAP-IG’s electronic version. These virtual means may have enormous potential in supporting/offering clinical care and/or in training personnel [47,48], including during and after the COVID-19 pandemic. We do acknowledge the importance of future research on implementing and using technology for delivering mental health care services, including exploring the barriers and facilitators of using technology to implement mhGAP-IG initiatives, as well as such technology’s effectiveness in comparison to using non-virtual means, as proposed by the Emilia project [31,32].

We experienced several challenges in conducting this review of the grey literature that are noteworthy to mention to generate further discussion on grey literature methodology, especially for the global health literature context. First, many of the documents did not have authors or dates. Neither did they all provide the name of the initiative that mobilized the mhGAP-IG. Thus, it was often difficult to associate authors with our findings to identify duplicates across platforms and to group documentation under an umbrella initiative. We opted to refrain from quantifying beyond an initial reporting on the number of retained documentation. Our review therefore aims to provide a qualitative and descriptive portrait of what we found. However, this grouping across search platforms, even if with only a couple of initiatives, could be an interesting exercise for future grey literature reviews, as it can help to improve understanding of how knowledge on implementation and use of the mhGAP-IG is being disseminated and through which platforms. Second, we noticed that information provided in the grey literature was heterogeneously reported within and across platforms. There was information that we originally planned to report in the findings but opted not to due to this inconsistency (ie, outcomes to comment on program effectiveness, number of non-specialists using the mhGAP-IG, number of training hours/days, number of people consulting mhGAP-based trained personnel, etc.). Lastly, we noticed that the content from some website links was sometimes inaccessible and/or no longer available, a challenge inherent in the grey literature: content and links may not be available as permanently as they are in peer-reviewed journals.

Limitations to conducting our review of the grey literature should also be noted. First, many of the initiatives came with references and additional resources. However, given limits to our research team, we opted not to review these references. These may have helped identify additional countries where the mhGAP-IG was implemented, and/or may have provided additional information on the mhGAP-IG’s use. Second, one of the hits identified on Google was the mhGAP hashtag on Twitter (#mhGAP). We did not search social media platforms such as Twitter and Facebook; however, searching these platforms may be promising avenues for future grey literature searches, given the increased use of social media for health related purposes in LMICs [49] and the ability to share information on mhGAP-IG implementation and use quickly and informally. Third, data extraction was conducted primarily by the first author, and this may have been subject to human error. Moreover, results on barriers and facilitators to the implementation and use of the mhGAP-IG were not always reported as such in the initiatives. Some barriers and facilitators were discussed as country/territory contextual factors, and the first author regrouped these as potential factors that may influence the implementation and use of the mhGAP-IG, a similar exercise explored elsewhere [50]. The identification of barriers and facilitators, as well as their grouping according to categories included in Chaudoir et al. (2013)’s [25] framework, may be subject to interpretation. The same should also be mentioned for the grouping of the outcome measures; these were done at the discretion of the first author. Nonetheless, all grouping of categories and synthesis of findings was reviewed by the second author and discussed at team meetings to help reduce bias and enhance clarity of the summaries. Fourth, it is worthy to note that some documents reported the use of the mhGAP-IG in parallel to other training material and/or initiatives, in the form of a package. For example, the PRIME project [51-54] relied on different-level initiatives, including the mhGAP-IG as a faculty-level support for providers to treat mental, neurological, and substance use disorders. Specifically, the mhGAP-IG was used as a part of mental health care plan packages. The specific use of the mhGAP-IG was sometimes difficult to tease out among these packages. Lastly, many of the grey literature sources we used to search for relevant materials were websites such as the MHIN website and the WHO website. Websites are subject to change in terms of features (eg, updates to their search engine), which is an important factor to consider for future replication or update of this review.

## CONCLUSIONS

Our overview of the grey literature provides rich experiential knowledge. Searching the grey literature may not only acknowledge the research and dissemination realities of many LMICs, but it can generate results that reinforce and/or expand peer-reviewed findings and discussion. We therefore encourage researchers conducting reviews on global health and global mental health topics to consider incorporating grey literature search strategies in their reviews.

## Additional material

Online Supplementary Document
