# Supplementary material for: Implementation and use of the Mental Health Gap Action Programme Intervention Guide (mhGAP-IG): A review of the grey literature
Source: J Glob Health. 2021 Apr 17;11:04022. doi: 10.7189/jogh.11.04022 (PMC8053394; doi:10.7189/jogh.11.04022)
Supplement: Online Supplementary Document [file jogh-11-04022-s001.pdf]

Online Supplementary Document
Table S1: Documents included in the review

Database	Number	Title	Link
**Proquest Dissertations & Theses Global**	1	Implicit and Explicit Attitudes of Medical Students Towards Mental Illness: A Randomized Controlled Pilot Trial of Service User Videos to Reduce Stigma in Nepal	https://search.proquest.com/pqdtglobal/docview/2046304344/C87173E1A4F64A51PQ/9?accountid=12543
**Database**		**Title**	**Link**
**Mental Health Innovation Network**	1	mhGAP 2.0 educational videos - need to be made!	https://www.mhinnovation.net/forums/mhin-africa-general-discussion-discussion-g%C3%A9n%C3%A9rale-discuss%C3%A3o-geral/mhgap-20-educational
	2	Stories of success at mhGAP Kashmir: What can we learn?	https://www.mhinnovation.net/blog/2017/sep/28/stories-success-mhgap-kashmir-what-can-we-learn
	3	Dreams do come true: How an mhGAP exchange project inspired a medical student to pursue a career in psychiatry	https://www.mhinnovation.net/blog/2017/sep/28/dreams-do-come-true-how-mhgap-exchange-project-inspired-medical-student-pursue
	4	Using mhGAP-IG on mobile phones for frontline healthcare workers to manage depression in Kenya	https://www.mhinnovation.net/innovations/using-mhgap-ig-mobile-phones-frontline-healthcare-workers-manage-depression-kenya
	5	Aro Primary Care Mental Health Programme	https://www.mhinnovation.net/innovations/aro-primary-care-mental-health-programme
	6	mhGAP Implementation in Kashmir	https://www.mhinnovation.net/innovations/mhgap-implementation-kashmir
	7	mhGAP Implementation in Lagos	https://www.mhinnovation.net/innovations/mhgap-implementation-lagos
	8	PRIME mhGAP in Ethiopia (Video)	https://www.mhinnovation.net/resources/prime-mhgap-ethiopia-video
	9	mhGAP Implementation in Edawu, Nigeria	https://www.mhinnovation.net/innovations/mhgap-implementation-edawu-nigeria
	10	Mental Health Awareness through mhGAP (Video)	https://www.mhinnovation.net/resources/mental-health-awareness-through-mhgap-video
	11	Teaching Skills Course/mhGAP Orientation	https://www.mhinnovation.net/forums/courses/teaching-skills-coursemhgap-orientation
	12	mhGAP in Ethiopia: Proof of Concept	https://www.mhinnovation.net/resources/mhgap-ethiopia-proof-concept
	13	mhGAP Intervention Guide: A systematic review of evidence from LMICs	https://www.mhinnovation.net/resources/mhgap-intervention-guide-systematic-review-evidence-lmics
	14	The FaNs for Kids Project	https://www.mhinnovation.net/innovations/fans-kids-project
	15	PRIME - Uganda	https://www.mhinnovation.net/innovations/prime-uganda
	16	Kenya integrated intervention model for dialogue and screening to promote children's mental wellbeing (KIDS)	https://www.mhinnovation.net/innovations/kenya-integrated-intervention-model-dialogue-and-screening-promote-childrens-mental-2

	17	Short Course: Summer Institute in Global Mental Health and Psychosocial Support (GMHPSS)	https://www.mhinnovation.net/forums/news-events/short-course-summer-institute-global-mental-health-and-psychosocial-support
	18	MHPSS Specialist CAR	https://www.mhinnovation.net/forums/vacancies-fellowships/mhpss-specialist-car
	19	MHPSS Specialist	https://www.mhinnovation.net/forums/vacancies-fellowships/mhpss-specialist
	20	MHPSS Specialist – Libya	https://www.mhinnovation.net/forums/vacancies-fellowships/mhpss-specialist-%E2%80%93-libya
	21	MHPSS Specialist 18-056	https://www.mhinnovation.net/forums/vacancies-fellowships/mhpss-specialist-18-056
	22	IRC Clinical Mental Health Specialist/Clinicien Spécialiste en Santé Mentale	https://www.mhinnovation.net/forums/vacancies-fellowships/irc-clinical-mental-health-specialistclinicien-sp%C3%A9cialiste-en-sant%C3%A9
	23	Scale-up and Evaluation of Family Networks for Children with Developmental Delays (FaNs for Kids)	https://www.mhinnovation.net/innovations/scale-and-evaluation-family-networks-children-developmental-delays-fans-kids
	24	PRIME - Nepal	https://www.mhinnovation.net/innovations/prime-nepal
	25	PRIME (PRogramme for Improving Mental health carE)	https://www.mhinnovation.net/innovations/prime-programme-improving-mental-health-care
	26	Training: Summer Institute in Global Mental Health and Psychosocial Support	https://www.mhinnovation.net/forums/news-events/training-summer-institute-global-mental-health-and-psychosocial-support
	27	Focus on Disability: Reshape mental healthcare in crises	https://www.mhinnovation.net/blog/2015/oct/20/focus-disability-reshape-mental-healthcare-crises
	28	PRIME Ethiopia	https://www.mhinnovation.net/innovations/prime-ethiopia
	29	PRIME India	https://www.mhinnovation.net/innovations/prime-india
	30	Where did all the depression go?	https://www.mhinnovation.net/blog/2014/dec/4/where-did-all-depression-go
	31	International Medical Corps: Psychiatrist Consultant, Chad	https://www.mhinnovation.net/forums/vacancies-fellowships/international-medical-corps-psychiatrist-consultant-chad
	32	Integrating MHPSS into Primary Health Care Centers in Lebanon	https://www.mhinnovation.net/innovations/integrating-mhpss-primary-health-care-centers-lebanon
	33	Partners In Health Liberia Mental Health Program	https://www.mhinnovation.net/innovations/partners-health-liberia-mental-health-program
	34	HSPR Summer School 2015: Global Mental Health - Research and Action	https://www.mhinnovation.net/forums/news-events/hspr-summer-school-2015-global-mental-health-research-and-action
	35	Mental Health & PsychoSocial Support Advisor, Save the Children UK (London) Deadline 4 Sept	https://www.mhinnovation.net/forums/vacancies-fellowships/mental-health-psychosocial-support-advisor-save-children-uk-london
	36	Establishing mental health and psychosocial support services during the Ebola virus disease outbreak in Sierra Leone	https://www.mhinnovation.net/innovations/establishing-mental-health-and-psychosocial-support-services-during-ebola-virus-disease
	37	Integration of mental health and psychosocial support services into primary healthcare in the Middle East	https://www.mhinnovation.net/innovations/integration-mental-health-and-psychosocial-support-services-primary-healthcare-middle
	38	MHPSS Specialist-IRC	https://www.mhinnovation.net/forums/vacancies-fellowships/mhpss-specialist-irc
	39	A mental health system strengthening consultant	https://www.mhinnovation.net/forums/vacancies-fellowships/mental-health-system-strengthening-consultant
	40	Mental Health Specialist – Cameroon	https://www.mhinnovation.net/forums/vacancies-fellowships/mental-health-specialist-%E2%80%93-cameroon

	41	Zanmi Lasante	https://www.mhinnovation.net/innovations/zanmi-lasante
	42	Primary Care 101+	https://www.mhinnovation.net/innovations/primary-care-101
	43	Improving mental health care for young people using e-health	https://www.mhinnovation.net/innovations/improving-mental-health-care-young-people-using-e-health
	44	Addressing Mental Health Needs of Children and their Families While Strengthening Health Systems	https://www.mhinnovation.net/addressing-mental-health-needs-children-and-their-families-while-strengthening-health-systems
	45	International Medical Corps MHPSS Trainer Libya	https://www.mhinnovation.net/forums/vacancies-fellowships/international-medical-corps-mhpss-trainer-libya
	46	Opportunity: HIPZ Mental Health Programme Volunteer	https://www.mhinnovation.net/forums/mhin-africa-opportunities-opportunit%C3%A9s-oportunidades/opportunity-hipz-mental-health-programme
	47	Healing invisible wounds of the Syrian conflict	https://www.mhinnovation.net/blog/2016/jan/19/healing-invisible-wounds-syrian-conflict
	48	Using mobile mental health clinics to expand access to services in post conflict rural areas	https://www.mhinnovation.net/innovations/using-mobile-mental-health-clinics-expand-access-services-post-conflict-rural-areas
	49	[Q&A Blog Series #MentalHealthAwareness Month] Mental Health Leadership in Africa	https://www.mhinnovation.net/blog/2018/may/8/qa-blog-series-mentalhealthawareness-month-mental-health-leadership-africa
	50	[#WHD2017 Africa Blog Series] Inspiring African innovations: Mental Health Scale Up Nigeria (mhSUN)	https://www.mhinnovation.net/blog/2017/apr/4/whd2017-africa-blog-series-inspiring-african-innovations-mental-health-scale-nigeria
	51	Mental Health Clinician and Supervisor-Institut des Soeurs Hospitalières de Notre Dame de la Compassion	https://www.mhinnovation.net/forums/mhin-africa-opportunities-opportunit%C3%A9s-oportunidades/mental-health-clinician-and-supervisor
	52	Psychiatrist Trainer – International Medical Corps – Central African Republic	https://www.mhinnovation.net/forums/vacancies-fellowships/psychiatrist-trainer-%E2%80%93-international-medical-corps-%E2%80%93-central-african
	53	Mental Health System Reform in Jordan	https://www.mhinnovation.net/innovations/mental-health-system-reform-jordan
	54	#studyGMH: student perspectives from the Centre for Global Mental Health	https://www.mhinnovation.net/blog/2015/apr/1/studygmh-student-perspectives-centre-global-mental-health
	55	Mental Health Beyond Facilities (mhBeF)	https://www.mhinnovation.net/innovations/mental-health-beyond-facilities-mhbef
	56	MHPSS Refugee Response in South Sudan	https://www.mhinnovation.net/innovations/mhpss-refugee-response-south-sudan
	57	International Medical Corps- MHPSS Coordinator Vacancy- Damascus, Syria	https://www.mhinnovation.net/forums/vacancies-fellowships/international-medical-corps-mhpss-coordinator-vacancy-damascus-syria
	58	[#WHD2017 Africa Blog Series] Inspiring African innovations: Aro Primary Care Mental Health Programme, Nigeria	https://www.mhinnovation.net/blog/2017/apr/4/whd2017-africa-blog-series-inspiring-african-innovations-aro-primary-care-mental
	59	Vacancy- MHPSS Specialist Cameroon International Medical Corps	https://www.mhinnovation.net/forums/vacancies-fellowships/vacancy-mhpss-specialist-cameroon-international-medical-corps
	60	Mental Health and Psychosocial Support Coordinator - Syria Response - International Medical Corps	https://www.mhinnovation.net/forums/vacancies-fellowships/mental-health-and-psychosocial-support-coordinator-syria-response
	61	Partners in Health Curriculum Toolkit	https://www.mhinnovation.net/resources/partners-health-curriculum-toolkit
	62	Psychotherapist/Trainer - Iraq - International Medical Corps	https://www.mhinnovation.net/forums/vacancies-fellowships/psychotherapisttrainer-iraq-international-medical-corps

	63	Putting a telepsychiatrist in communities in the Union of Comoros	https://www.mhinnovation.net/innovations/putting-telepsychiatrist-communities-union-comoros
	64	[#WHD2017 Blog Series] Inspiring Innovations: MHPSS in the Middle East	https://www.mhinnovation.net/blog/2017/apr/7/whd2017-blog-series-inspiring-innovations-mhpss-middle-east
	65	Mental health in Nepal: Time to invest more on the sector	https://www.mhinnovation.net/blog/2018/jul/11/mental-health-nepal-time-invest-more-sector
	66	Psychiatrist – Roster for Emergency Response	https://www.mhinnovation.net/forums/vacancies-fellowships/psychiatrist-%E2%80%93C2%A0roster-emergency-response
	67	Family Well-being Centers: Delivering Community-based Mental Health Support in Syria	https://www.mhinnovation.net/innovations/family-well-being-centers-delivering-community-based-mental-health-support-syria
	68	MIND ME Africa	https://www.mhinnovation.net/innovations/mind-me-africa
	69	Infographic: Innovations implementing the WHO Mental Health Gap Action Programme (mcGAP) Intervention Guide	https://www.mhinnovation.net/resources/infographic-innovations-implementing-who-mental-health-gap-action-programme-mhgap
	70	Facilitating Mental Health Awareness and Community Engagement in Sierra Leone	https://www.mhinnovation.net/innovations/facilitating-mental-health-awareness-and-community-engagement-sierra-leone
	71	SMART Mental Health	https://www.mhinnovation.net/innovations/smart-mental-health
	72	Comprehensive Community Mental Health Programme	https://www.mhinnovation.net/innovations/comprehensive-community-mental-health-programme
	73	COM-GAP-S: Integrating mental health into primary care for post-conflict populations in Northern Sri Lanka	https://www.mhinnovation.net/innovations/com-gap-s-integrating-mental-health-primary-care-post-conflict-populations-northern-sri
	74	Mental Health Scale Up Nigeria (mhSUN)	https://www.mhinnovation.net/innovations/mental-health-scale-nigeria-mhsun
**Database**		**Title**	**Link**
**mhGAP Newsletters**	1	The Intervention Guide will help scale-up care for mental, neurological and substance use disorders (Jan. 2011)	https://www.who.int/mental_health/mhGAP_newsletter_jan2011.pdf
	2	Partners commit to mhGAP at the Forum meeting (Jan. 2011)	https://www.who.int/mental_health/mhGAP_newsletter_jan2011.pdf
	3	Partners report progress (Dec. 2011)	https://www.who.int/mental_health/mhgap/mhGAP_nl_december_2011.pdf
	4	Training gurus from all over the world meet in Italy (Dec. 2011)	https://www.who.int/mental_health/mhgap/mhGAP_nl_december_2011.pdf
	5	News from WHO's Mental Health Gap Action Programme (mhGAP) (June 2011)	https://www.who.int/mental_health/mhGAP_nl__jun2011_en.pdf
	6	European Commission funding enables scaling up of mental health services in Ethiopia and Nigeria (June 2011)	https://www.who.int/mental_health/mhGAP_nl__jun2011_en.pdf
	7	Ethiopian Government commits to mhGAP implementation (June 2011)	https://www.who.int/mental_health/mhGAP_nl__jun2011_en.pdf
	8	Panama mhGAP projet launch (June 2011)	https://www.who.int/mental_health/mhGAP_nl__jun2011_en.pdf

	9	Nigeria integrates mental health into primary health care (June 2011)	https://www.who.int/mental_health/mhGAP_nl__jun2011_en.pdf
	10	Jordan starts training primary care doctors and nurses using mhGAP Intervention Guide (June 2011)	https://www.who.int/mental_health/mhGAP_nl__jun2011_en.pdf
	11	Update from Partners (June 2011)	https://www.who.int/mental_health/mhGAP_nl__jun2011_en.pdf
	12	mhGAP takes off in Uganda (June 2012)	
	13	Sierra Leone nurses: pioneers in mental health (June 2012)	https://www.who.int/mental_health/mhGAP_nl_June_2012.pdf
	14	mhGAP training saves a life in Honduras (June 2012)	https://www.who.int/mental_health/mhGAP_nl_June_2012.pdf
	15	mhGAP base training course – now available for field testing (June 2012)	https://www.who.int/mental_health/mhGAP_nl_June_2012.pdf
	16	mhGAP Scaling up mental health services in the Eastern Mediterranean Region (June 2013)	https://www.who.int/mental_health/mhgap/mhGAP_nl_June_2013.pdf
	17	Mental health training: planting the seed early could make a difference (June 2013)	https://www.who.int/mental_health/mhgap/mhGAP_nl_June_2013.pdf
	18	WHO responds to mental health in emergencies (Dec. 2013)	https://www.who.int/mental_health/mhgap/mhGAP_nl_December_2013.pdf
	19	mhGAP training can change lives (Dec. 2013)	https://www.who.int/mental_health/mhgap/mhGAP_nl_December_2013.pdf
	20	Reaching the unreached in resource-poor settings in Francophone countries (Dec. 2013)	https://www.who.int/mental_health/mhgap/mhGAP_nl_December_2013.pdf
	21	From virtual to practical: mhGAP in the Caribbean (June 2014)	https://www.who.int/mental_health/mhgap/Newsletter_June_2014.pdf
	22	Ethiopia commits to expanding mental health services (June 2014)	https://www.who.int/mental_health/mhgap/Newsletter_June_2014.pdf
	23	Community: a key ingredient to mental health recovery (Jan. 2015)	https://www.who.int/mental_health/mhgap/story_somalia_2014/en/
	24	Collaboration benefits mhGAP training in Mexico (Jan. 2015)	https://www.who.int/mental_health/mhgap/mexico_story_2014/en/
	25	Ethiopia's first National Mental Health Symposium calls for reform (Jan. 2015)	https://www.who.int/mental_health/mhgap/national_mh_symposium_ethiopia/en/
	26	mhGAP training creates more support for vulnerable people in Syria (July 2015)	https://www.who.int/mental_health/mhgap/syria_story/en/
	27	mhGAP: supporting Ebola survivors in Guinea (Jan. 2016)	https://www.who.int/mental_health/mhgap/guinea_ebola/en/
	28	Scale up of mhGAP across a disaster-affected region in the Philippines (Jan. 2016)	https://www.who.int/mental_health/emergencies/mhgap_philippines/en/
	29	Mainstreaming mental health in Ethiopia (May 2016)	https://www.who.int/mental_health/mhgap/ethiopia_story_2016/en/
	30	Building general practitioner capacity in Tunisia by implementing the mhGAP (May 2016)	https://www.who.int/mental_health/mhgap/mhgap_tunisia/en/

	31	Continuing momentum in reforming access to care in West Africa (Nov. 2016)	https://www.who.int/mental_health/mhgap/cbm_care_west_africa/en/
	32	mhGAP in Uganda – bringing treatment, dignity and real change (Nov. 2016)	https://www.who.int/mental_health/mhgap/uganda_world_vision/en/
	33	Collaboration – for strengthening mental health services in Zimbabwe (Nov. 2016)	https://www.who.int/mental_health/mhgap/zimbabwe/en/
	34	Bringing mental health care to conflict-affected Ukraine (Nov. 2016)	https://www.who.int/mental_health/emergencies/mental_health_Ukraine/en/
	35	Suicide prevention in Guyana (March 2017)	https://www.who.int/mental_health/suicide-prevention/guyana/en/
	36	Health staff in the Central African Republic trained on mental disorders (July 2017)	https://www.who.int/mental_health/mhgap/CAR_2017/en/
	37	Strengthening mental health services in Cox's Bazar (April 2018)	https://mailchi.mp/who/who-mhgap-newsletter-april-2018?e=a3f1907369
	38	Access to mental health care increases in Chile through mhGAP training programme (April 2018)	https://www.paho.org/hq/index.php?option=com_content&view=article&id=14145:chile-mhgap-training-of-primary-care-teams&Itemid=42050&lang=en
	39	Foundation for provision of mental health and psychosocial support in South Sudan strengthened (Nov. 2018)	https://mailchi.mp/who/who-mhgap-newsletter-november-2018
	40	Mental health services for Syrian refugees in Turkey strengthened (Nov. 2018)	https://mailchi.mp/who/who-mhgap-newsletter-november-2018
	41	Integrated mental healthcare approach shows success (April 2019)	https://www.who.int/mental_health/mhgap/PRIME/en/
	42	Mental health care accessible in primary care level in Eastern Visayas (April 2019)	https://www.who.int/philippines/news/feature-stories/detail/mental-health-care-accessible-at-the-primary-level-in-eastern-visayas
	43	Building capacity for mental health care in Eastern Europe and Central Asia (April 2019)	https://www.who.int/mental_health/mhgap/eastern_europe_central_asia/en/
**Database**		**Title**	**Link**
**WHO website**	1	Scaling Up Services for Mental, Neurological, and Substance Use Disorder in Ethiopia	https://www.who.int/mental_health/mhgap/mhgap_ethiopia_brochure.pdf
	2	Syrian Arab Republic Situation reports	https://www.who.int/hac/crises/syr/sitreps/en/
	3	Narrowing mental health gaps one year after Typhoon Yolanda	https://www.who.int/philippines/news/feature-stories/detail/narrowing-mental-health-gaps-one-year-after-typhoon-yolanda
	4	WHO-AIMS Report on Mental Health Systems in Jordan	https://www.who.int/mental_health/evidence/mh_aims_report_jordan_jan_2011_en.pdf
	5	Zimbabwe commemorates World Mental Health Day	https://www.afro.who.int/news/zimbabwe-commemorates-world-mental-health-day?country=883&name=Zimbabwe
	6	WHO: health humanitarian appeal for the Rohingya crisis 2018	https://www.who.int/emergencies/crises/bgd/bangladesh-humanitarian-appeal-rohingya-crisis-2018.pdf
	7	Mental Health Gap Action Programme (mhGAP) 2nd Meeting of the mhGAP Forum	https://www.who.int/mental_health/mhgap/mhgap_forum_oct2010_annex_f.pdf

	8	Mental Health Gap Action Programme (mhGAP) 3rd meeting of the mhGAP Forum	https://www.who.int/mental_health/mhgap/mhgap_forum_oct2011_annex_e.pdf
	9	WHO and Ministry of Health conduct mental health training for non-specialist health care professionals in Al-Anbar governorate	http://www.emro.who.int/irq/iraq-news/who-and-ministry-of-health-conduct-mental-health-training-for-non-specialist-health-care-professionals-in-al-anbar-governorate.html
	10	WHO Liberia 2016 Annual Report	https://www.afro.who.int/sites/default/files/2017-12/WHO%20Liberia%20Annual%20Report%202016_2.pdf
	11	Joint Mission of the United Nations Interagency Task Force on the Prevention and Control of Non-Communicable Diseases	https://www.afro.who.int/sites/default/files/2019-01/UNIATF%20mission%20in%20ETHIOPIA_18MAY2018%20(1)%20(2).pdf
	12	Annual Report of the Director of the Pan American Sanitary Bureau (57th Directing Council)	https://www.paho.org/hq/index.php?option=com_docman&view=download&alias=49816-cd57-3-e-annual-report-director&category_slug=cd57-en&Itemid=270&lang=en
	13	Annual Report of the Director of the Pan American Sanitary Bureau (56th Directing Council)	https://www.paho.org/hq/index.php?option=com_docman&view=download&alias=46223-cd56-3-e-report-director&category_slug=56-directing-council-english-9964&Itemid=270&lang=en
	14	Quinquennial Report of the Director of the Pan American Sanitary Bureau	https://reliefweb.int/sites/reliefweb.int/files/resources/9789275119709_eng.pdf
	15	Quinquennial Report 2013 – 2017 of the Director of the Pan American Sanitary Bureau Championing Health for Sustainable Development and Equity: On the Road to Universal Health	https://www.paho.org/annual-report-2017/index.html
	16	Progress Reports on Technical Matters	https://www.paho.org/hq/index.php?option=com_docman&view=download&category_slug=29-en-9249&alias=41932-csp29-inf-7-e-932&Itemid=270&lang=en
	17	Epilepsy in Latin America	https://www.paho.org/hq/dmdocuments/2016/Epilepsy-in-Latin-America.pdf
	18	Regional Situation Report, April 2015 WHO response to the Syrian crisis	http://www.emro.who.int/images/stories/syria/WHO_SitRep_April2015.pdf
**Database**		**Title**	**Link**
**Google**	1	Using Mobile Phones to Empower Frontline Healthcare Workers to Manage Depression at Point of Care in Kenya Using the WHO Mental Health Treatment Gap Intervention Guidelines (mhGAP-IG)	https://www.grandchallenges.ca/grantee-stars/0414-01/
	2	Mental Health Gap Action (mhGAP) Virtual Course 2017	https://www.campusvirtualsp.org/sites/default/files/webfiles/onlineflyers/cvmhgap17/cvmhgap17.html
	3	WHO: Mental Health Gap Action Programme - YouTube	https://www.youtube.com/watch?v=TqlafjsOaoM
	4	Building capacity by implementing mhGAP mobile intervention in SADC countries	https://mega.turkuamk.fi/our-project/
	5	Orientation WHO mhGAP Intervention Guide V2 & Psychological First Aid	https://www.eventbrite.co.uk/e/orientation-who-mhgap-intervention-guide-v2-psychological-first-aid-tickets-73125156311

	6	International Alliance of Women	https://womenalliance.org/iaw-launches-marathi-translation-of-world-health-organizations-mental-health-gap-action-programme-intervention-guide-mhgap-ig
	7	E-mhGAP Intervention guide in Low and middle-income countries: proof-of-concept for Impact and Acceptability (Emilia)	http://tponepal.org/e-mhgap-intervention-guide-in-low-and-middle-income-countries-proof-of-concept-for-impact-and-acceptability-emilia/
	8	E-MhGAP Intervention guide in Low and middle income countries: proof-of-concept for Impact and Acceptability (Emilia project)	https://gtr.ukri.org/projects?ref=MR%2FS001255%2F1
	9	mhGAP forum: Mental health capacity building within countries	https://www.fondationdharcourt.org/mhgap-forum-2017/
	10	Mental Health Programme mhGAP Provides Healthcare Access to IDPS	https://borgenproject.org/mhgap-healthcare-nigeria/
	11	Assessing the efficacy of the Mental Health Gap Action Programme (mhGAP) training for non-specialized health workers in Ethiopia	https://run.unl.pt/bitstream/10362/13220/1/Bruni%20Andrea%20TM%202014.pdf
	12	PRIME at the WHO mhGAP Forum	http://www.prime.uct.ac.za/news/prime-who-mhgap-forum
	13	Another step towards mental health for all	https://www.cbm.org/news/news/news-2016/another-step-towards-mental-health-for-all/
	14	GP clinics could help bridge mental health treatment gap, study finds	https://www.gatescambridge.org/about/news/gp-clinics-could-help-bridge-mental-health-treatment-gap-study-finds/
	15	Six years after ‘Yolanda,’ mental scars linger	https://newsinfo.inquirer.net/1187193/6-years-after-yolanda-mental-scars-linger

Table S2: Country implementation, according to World Bank Income Categories and World Health Organization Regions

Country/Territory of Implementation	World Bank Income Categories	World Health Organization (WHO) Region	Adaptation
Afghanistan	Low-income	WHO Eastern Mediterranean Region	x
Albania	Upper-middle-income	WHO European Region	
Argentina	Upper-middle-income	WHO Region of the Americas	
Bahamas	High-income	WHO Region of the Americas	
Bangladesh	Lower-middle-income	WHO South-East Asia Region	
Belize	Upper-middle-income	WHO Region of the Americas	
Benin	Lower-middle-income	WHO African Region	x
Bolivia	Lower-middle-income	WHO Region of the Americas	
Brazil	Upper-middle-income	WHO Region of the Americas	x
British Virgin Islands	High-income	Not available	
Burkina Faso	Low-income	WHO African Region	x
Cameroon	Lower-middle-income	WHO African Region	
Central African Republic	Low-income	WHO African Region	x
Chad	Low-income	WHO African Region	x
Chile	High-income	WHO Region of the Americas	
China (rural)	Upper-middle-income	WHO Western Pacific Region	
Colombia	Upper-middle-income	WHO Region of the Americas	
Costa Rica	Upper-middle-income	WHO Region of the Americas	
Cuba	Upper-middle-income	WHO Region of the Americas	
Dominica	Upper-middle-income	WHO Region of the Americas	
Egypt	Lower-middle-income	WHO Eastern Mediterranean Region	
El Salvador	Lower-middle-income	WHO Region of the Americas	
Ethiopia	Low-income	WHO African Region	x
Federated States of Micronesia	Lower-middle-income	WHO Western Pacific Region	
Ghana	Lower-middle-income	WHO African Region	
West Bank and Gaza	Lower-middle-income	Not available	
Georgia	Upper-middle-income	WHO European Region	
Greece	High-income	WHO European Region	x

Grenada	Upper-middle-income	WHO Region of the Americas	
Guatemala	Upper-middle-income	WHO Region of the Americas	
Guinea	Low-income	WHO African Region	
Guyana	Upper-middle-income	WHO Region of the Americas	
Haiti	Low-income	WHO Region of the Americas	x
Honduras	Lower-middle-income	WHO Region of the Americas	
Iraq	Upper-middle-income	WHO Eastern Mediterranean Region	x
India	Lower-middle-income	WHO South-East Asia Region	x
Indonesia	Upper-middle-income	WHO South-East Asia Region	
Italy	High-income	WHO European Region	x
Jamaica	Upper-middle-income	WHO Region of the Americas	
Japan	High-income	WHO Western Pacific Region	
Jordan	Upper-middle-income	WHO Eastern Mediterranean Region	x
Kenya	Lower-middle-income	WHO African Region	x
Lebanon	Upper-middle-income	WHO Eastern Mediterranean Region	x
Lesotho	Lower-middle-income	WHO African Region	
Liberia	Low-income	WHO African Region	
Libya	Upper-middle-income	WHO Eastern Mediterranean Region	x
Marshall Islands	Upper-middle-income	WHO Western Pacific Region	
Mexico	Upper-middle-income	WHO Region of the Americas	x
Mozambique	Low-income	WHO African Region	
Myanmar	Lower-middle-income	WHO South-East Asia Region	
Nepal	Lower-middle-income	WHO South-East Asia Region	x
Nicaragua	Lower-middle-income	WHO Region of the Americas	
Niger	Low-income	WHO African Region	x
Nigeria	Lower-middle-income	WHO African Region	x
Norway	High-income	WHO European Region	
Pakistan	Lower-middle-income	WHO Eastern Mediterranean Region	x
Panama	High-income	WHO Region of the Americas	x
Papua New Guinea	Lower-middle-income	WHO Western Pacific Region	
Peru	Upper-middle-income	WHO Region of the Americas	
Philippines	Lower-middle-income	WHO Region of the Americas	

Côte d'Ivoire	Lower-middle-income	WHO African Region	x
Republic of Palau	High-income	WHO Region of the Americas	
Saint Kitts and Nevis	High-income	WHO Region of the Americas	
Saint Vincent and the Grenadines	Upper-middle-income	WHO Region of the Americas	
Sierra Leone	Low-income	WHO African Region	
Sint Maarten	High-income	Not available	
Somalia	Low-income	WHO Eastern Mediterranean Region	
Somaliland	Not available	Not available	
South Africa	Upper-middle-income	WHO African Region	x
South Sudan	Low-income	WHO African Region	
Spain	High-income	WHO European Region	x
Sri Lanka	Lower-middle-income	WHO South-East Asia Region	x
Suriname	Upper-middle-income	WHO Region of the Americas	
Syrian Arab Republic	Low-income	WHO Eastern Mediterranean Region	x
Thailand	Upper-middle-income	WHO South-East Asia Region	
The Gambia	Low-income	WHO African Region	
Trinidad and Tobago	High-income	WHO Region of the Americas	
Togo	Low-income	WHO African Region	
Turkey	Upper-middle-income	WHO European Region	x
Turkmenistan	Upper-middle-income	WHO European Region	
Turks and Caicos Islands	High-income	Not available	
Tunisia	Lower-middle-income	WHO Eastern Mediterranean Region	
Uganda	Low-income	WHO African Region	x
Ukraine	Lower-middle-income	WHO European Region	x
Union of Comoros	Lower-middle-income	WHO African Region	
United Kingdom	High-income	WHO European Region	
United States of America	High-income	WHO Region of the Americas	
Venezuela	Upper-middle-income	WHO Region of the Americas	
Vietnam	Lower-middle-income	WHO Western Pacific Region	
Zambia	Lower-middle-income	WHO African Region	
Zanzibar	Lower-middle-income	WHO African Region	
Zimbabwe	Lower-middle-income	WHO African Region	x
